# Supplementary figures and images for: Insights on Pinna nobilis population genetic structure in the Aegean and Ionian Sea
Source: PeerJ. 2023 Nov 29;11:e16491. doi: 10.7717/peerj.16491 (PMC10693241; doi:10.7717/peerj.16491)

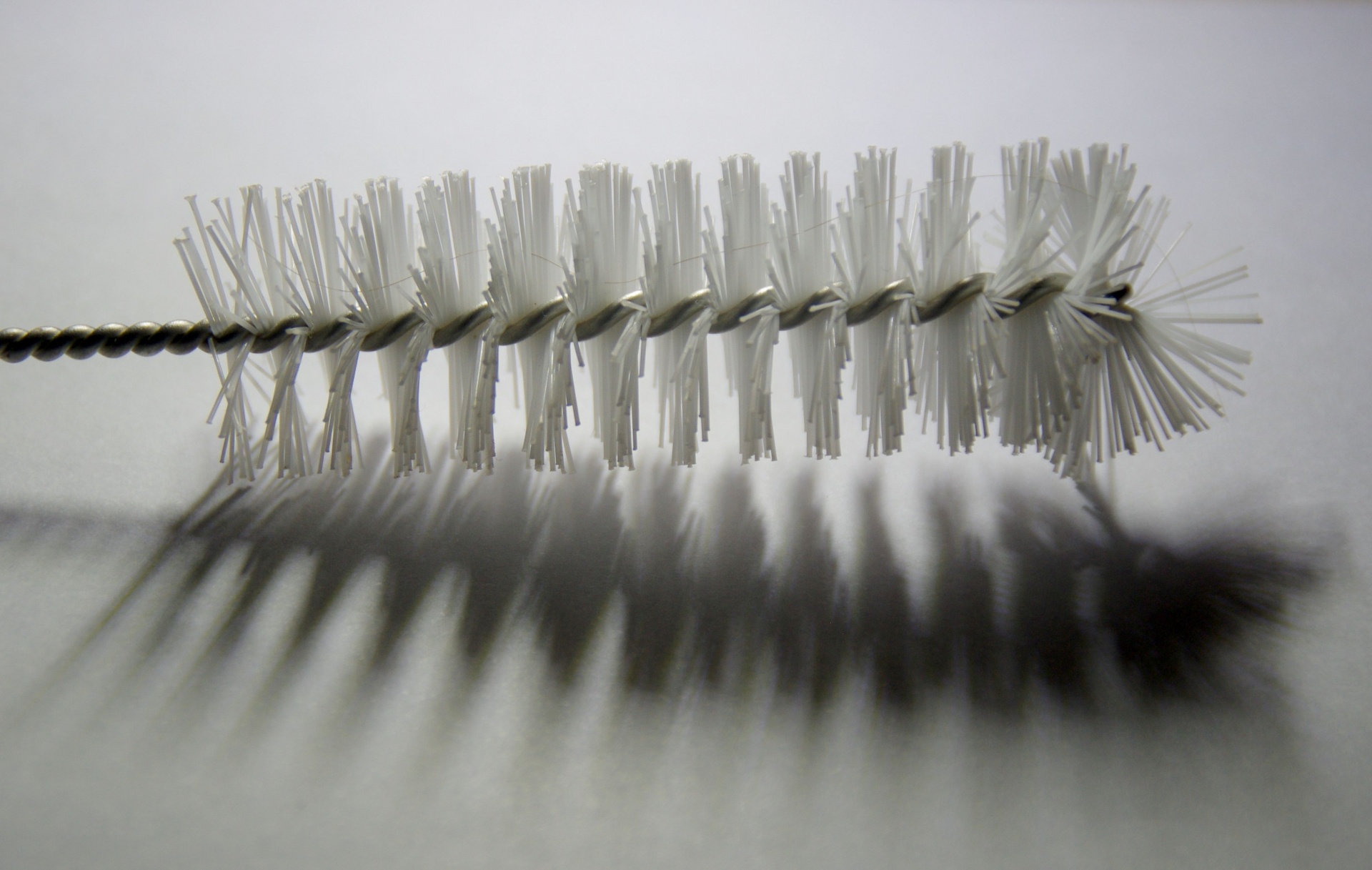

Supplement: Supplemental Information 1 [file peerj-11-16491-s001.jpg]

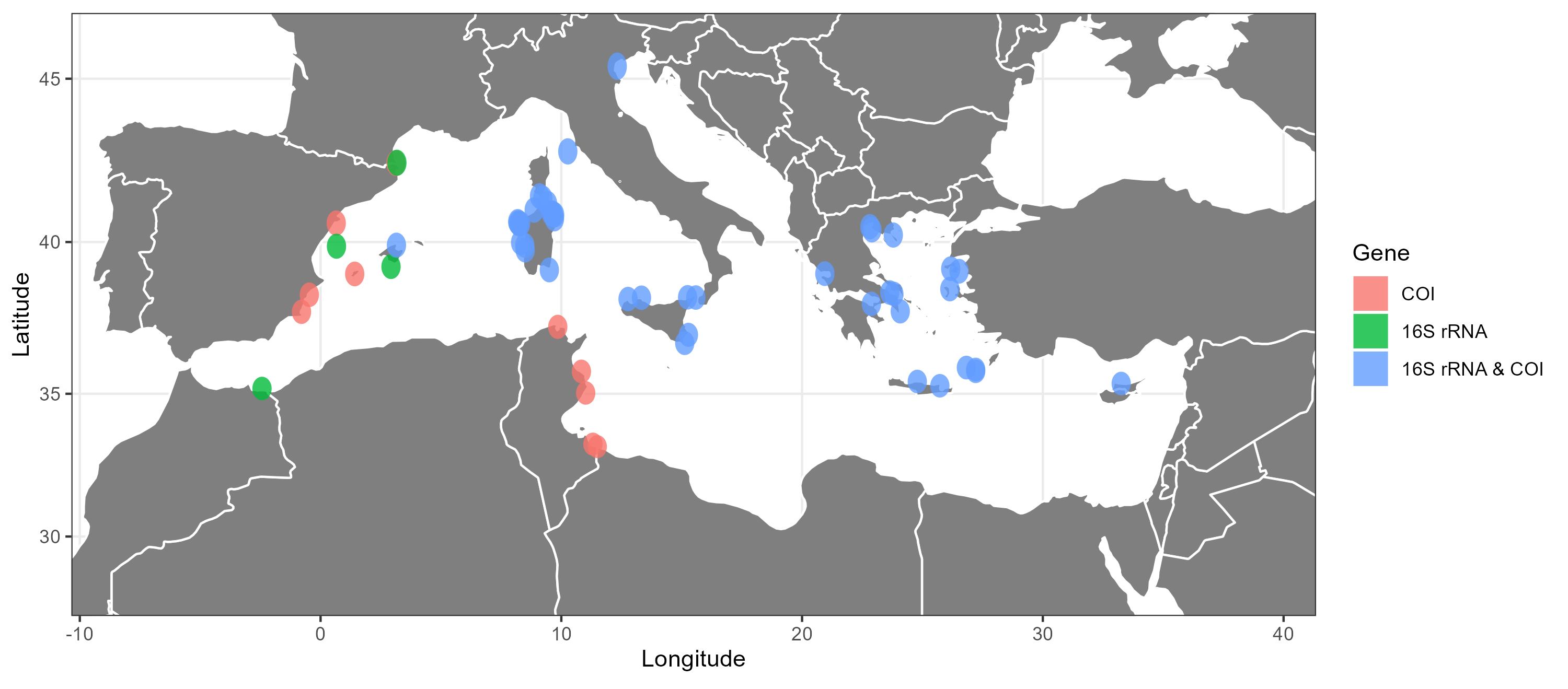

Supplement: Supplemental Information 2 [file peerj-11-16491-s002.png]

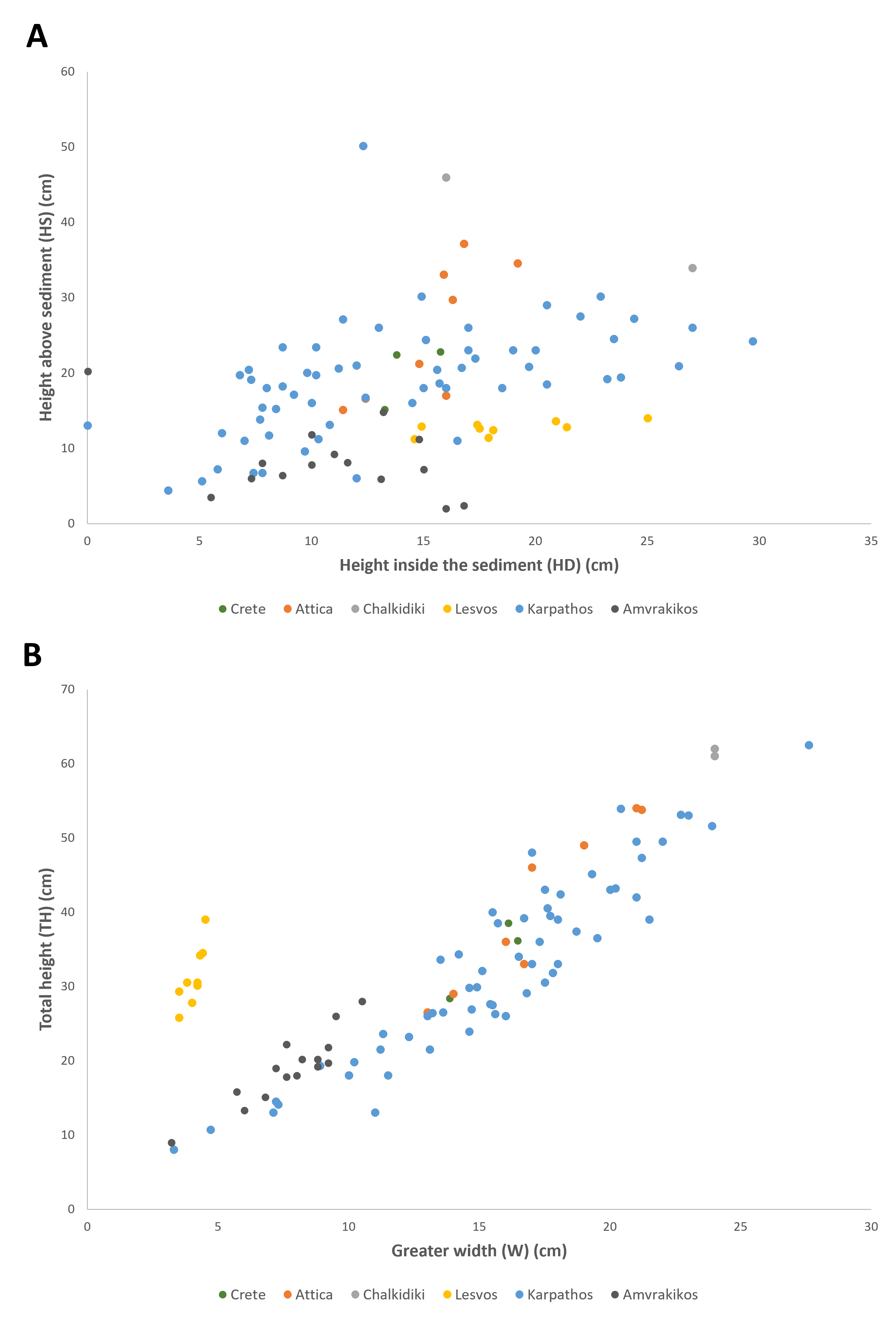

Supplement: Supplemental Information 3 — (A) Scatterplot showing the relationship between the shell’s height above sediment with the shell’s height buried inside the sediment for the P. nobilis individuals sampled for this study. (B) Scatterplot showing the relationship between the shell’s total height and the shell’s width for the P. nobilis individuals sampled for this study. [file peerj-11-16491-s003.png]

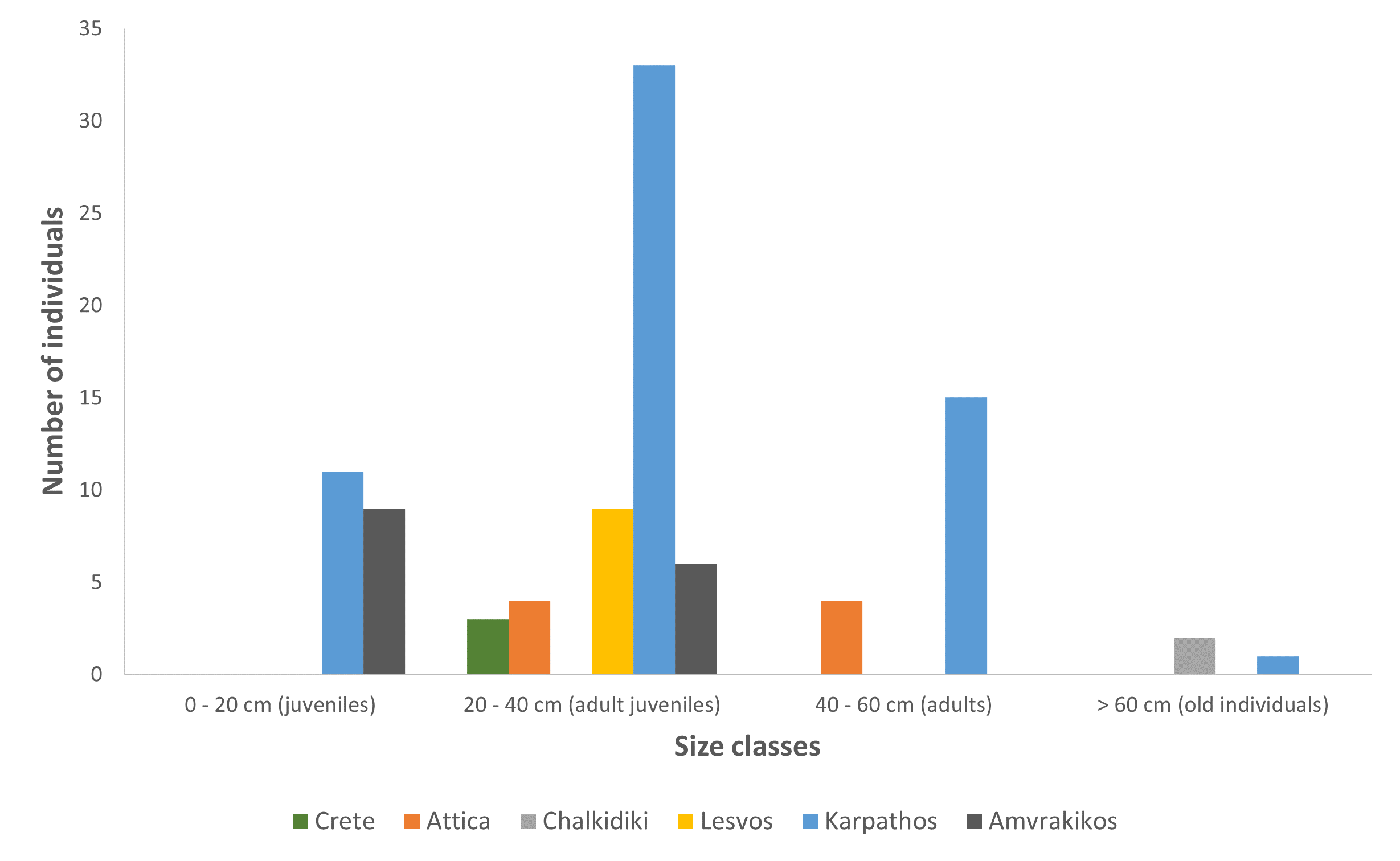

Supplement: Supplemental Information 4 — The shell’s total height distribution categorized in age classes for the different individuals sampled for this study. [file peerj-11-16491-s004.png]

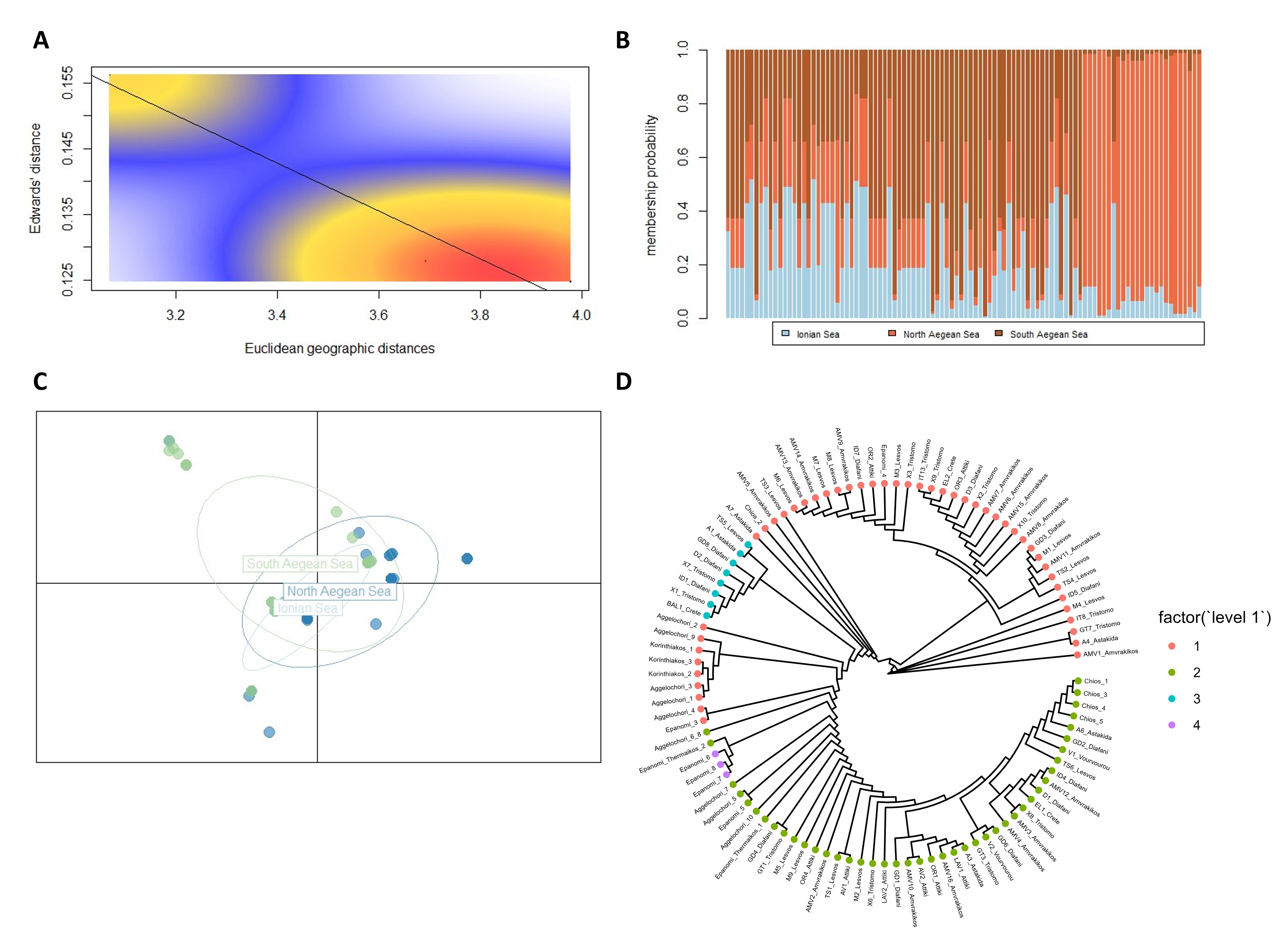

Supplement: Supplemental Information 5 — (A) Isolation by distance scatterplot for the 16S rRNA-COI Greece (Eastern Mediterranean) dataset. (B) Membership probability of individuals to populations, as calculated by DAPC, for the 16S rRNA-COI Greece (Eastern Mediterranean) dataset. (C) PCA plot depicting the similarity of different populations for the 16S rRNA-COI Greece (Eastern Mediterranean) dataset. (D) Phylogenetic tree of the 16S rRNA-COI Greece (Eastern Mediterranean) dataset showing the hierBAPS clustering into different groups. [file peerj-11-16491-s005.png]

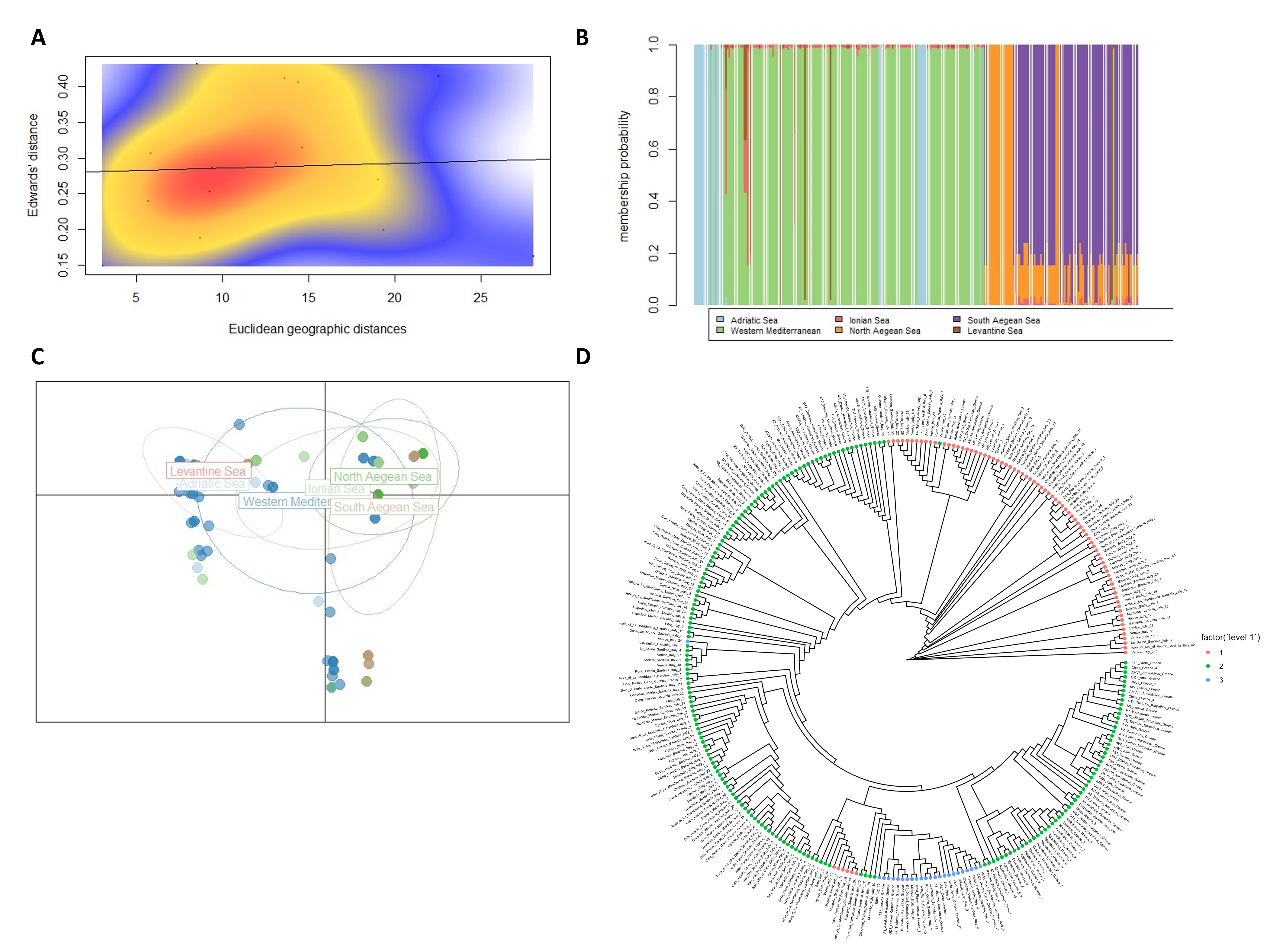

Supplement: Supplemental Information 6 — (A) Isolation by distance scatterplot for the 16S rRNA-COI Central, Western and Eastern Mediterranean Sea dataset. (B) Membership probability of individuals to populations, as calculated by DAPC, for the 16S rRNA-COI Central, Western and Eastern Mediterranean Sea dataset. (C) PCA plot depicting the similarity of different populations for the 16S rRNA-COI Central, Western and Eastern Mediterranean Sea dataset. (D) Phylogenetic tree of the 16S rRNA-COI Central, Western and Eastern Mediterranean Sea dataset showing the hierBAPS clustering into different groups. [file peerj-11-16491-s006.png]

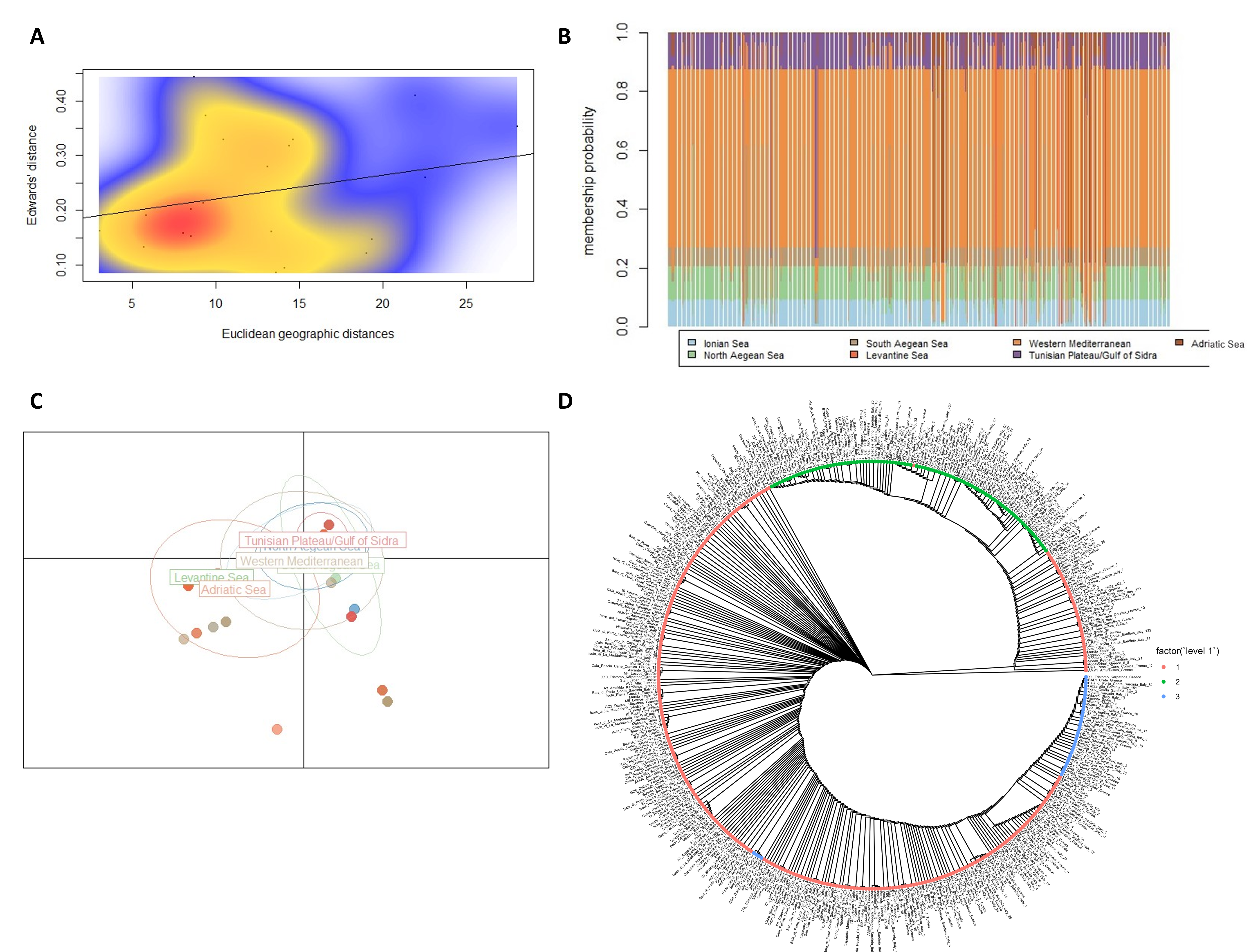

Supplement: Supplemental Information 7 [file peerj-11-16491-s007.png]
