# Supplementary material for: Insights on Pinna nobilis population genetic structure in the Aegean and Ionian Sea
Source: PeerJ. 2023 Nov 29;11:e16491. doi: 10.7717/peerj.16491 (PMC10693241; doi:10.7717/peerj.16491)
Supplement: Supplemental Information 8 — TH: Total height, HS: Height above sediment, HD: Height inside the sediment, W: Greater width, DNA conc: DNA concentration of the samples (ng/µl); where DNA was extracted for multiple tissues, the average DNA concentration is shown. [file peerj-11-16491-s008.docx]

| Supplementary Table 1: Details and metadata of the samples. TH: Total height, HS: Height above sediment, HD: Height inside the sediment, W: Greater width, DNA conc: DNA concentration of the samples (ng/µl); where DNA was extracted for multiple tissues, the average DNA concentration is shown. | | | | | | | | | | | |  |
| --- | --- | --- | --- | --- | --- | --- | --- | --- | --- | --- | --- | --- |
| **Sample code** | **Location** | **Latitude/Longitude** | **Collection date** | **Sampling method** | **16S rRNA accession number** | **COI accession number** | **Depth (m)** | **TH (cm)** | **HS (cm)** | **HD (cm)** | **W (cm)** | **DNA conc.** |
| EL01 | Elounda (Crete) | 35.272369/25.723469 | 12/2/2019 | tissue | OX406991 | OX407172 | 3 | 38.55 | 22.8 | 15.75 | 16.1 | 2071.56 |
| EL02 | Elounda (Crete) | 35.272389/25.723472 | 12/2/2019 | tissue | OX406992 | OX407173 | 3 | 36.19 | 22.4 | 13.79 | 16.45 | 2396.72 |
| BAL01 | Bali (Crete) | 35.416267/24.785967 | 6/3/2019 | tissue | OX406993 | OX407174 | 5 | 28.4 | 15.15 | 13.25 | 13.85 | 1164.91 |
| AV01 | Avlida (Attica) | 38.373219/23.640273 | 3/3/2019 | tissue | OX406994 | OX407175 | 1.5 | 46 | 29.7 | 16.3 | 17 | 973.33 |
| AV02 | Avlida (Attica) | 38.373219/23.640273 | 3/3/2019 | tissue | OX406995 | OX407176 | 1.5 | 36 | 21.2 | 14.8 | 16 | 932.91 |
| OR01 | Oropos (Attica) | 38.328049/23.807744 | 8/3/2019 | tissue | OX406996 | OX407177 | 3.4 | 29 | 16.6 | 12.4 | 14 | 1822.52 |
| OR02 | Oropos (Attica) | 38.328049/23.807744 | 8/3/2019 | tissue | OX406997 | OX407178 | 4.3 | 26.5 | 15.1 | 11.4 | 13 | 2990.83 |
| OR03 | Oropos (Attica) | 38.320699/23.820207 | 8/3/2019 | tissue | OX406998 | OX407179 | 3.8 | 54 | 37.2 | 16.8 | 21 | 1960.08 |
| OR04 | Oropos (Attica) | 38.320699/23.820207 | 8/3/2019 | tissue | OX406999 | OX407180 | 3.6 | 49 | 33.1 | 15.9 | 19 | 1898.10 |
| VOUR01 | Vourvourou (Chalkidiki) | 40.221874/23.788816 | 28/4/2019 | tissue | OX407000 | OX407181 | 7 | 61 | 34 | 27 | 24 | 451.90 |
| VOUR02 | Vourvourou (Chalkidiki) | 40.221874/23.788816 | 28/4/2019 | tissue | OX407001 | OX407182 | 7 | 62 | 46 | 16 | 24 | 249.03 |
| Α1 | Astakida (Karpathos) | 35.886497/26.824323 | 10/7/2018 | eDNA | OX407002 | OX407183 | 10 | 19.3 | 9.6 | 9.7 | 8.9 | 89.34 |
| Α2 | Astakida (Karpathos) | 35.886497/26.824323 | 10/7/2018 | eDNA | -- | -- | 10 | 19.8 | 11.7 | 8.1 | 10.2 | 337.08 |
| Α3 | Astakida (Karpathos) | 35.886497/26.824323 | 10/7/2018 | eDNA | OX407003 | OX407184 | 10 | 21.5 | 13.8 | 7.7 | 11.2 | 49.77 |
| Α4 | Astakida (Karpathos) | 35.886497/26.824323 | 10/7/2018 | eDNA | OX407004 | OX407185 | 10 | 14.5 | 6.7 | 7.8 | 7.2 | 121.07 |
| Α5 | Astakida (Karpathos) | 35.886497/26.824323 | 10/7/2018 | eDNA | -- | -- | 10 | 10.7 | 5.6 | 5.1 | 4.7 | 111.95 |
| Α6 | Astakida (Karpathos) | 35.886497/26.824323 | 10/7/2018 | eDNA | OX407005 | OX407186 | 10 | 13 | 7.2 | 5.8 | 7.1 | 136.30 |
| Α7 | Astakida (Karpathos) | 35.886497/26.824323 | 10/7/2018 | eDNA | OX407006 | OX407187 | 10 | 14.1 | 6.7 | 7.4 | 7.3 | 145.27 |
| AMV1 | Amvrakikos Gulf | 38.9856717451461/20.94545047741468 | 17/4/2021 | tissue | OX407016 | OX407196 | -- | 18 | 2 | 16 | 8 | 1518.77 |
| AMV2 | Amvrakikos Gulf | 38.9856717451461/20.94545047741468 | 17/4/2021 | tissue | OX407017 | OX407197 | -- | 19.2 | 2.4 | 16.8 | 8.8 | 1319.00 |
| AMV3 | Amvrakikos Gulf | 38.9856717451461/20.94545047741468 | 17/4/2021 | tissue | OX407018 | OX407198 | -- | 20.2 | 20.2 | 0 | 8.2 | 1189.04 |
| AMV4 | Amvrakikos Gulf | 38.9856717451461/20.94545047741468 | 17/4/2021 | tissue | OX407019 | OX407199 | -- | -- | -- | -- | -- | 476.10 |
| AMV5 | Amvrakikos Gulf | 38.9856717451461/20.94545047741468 | 17/4/2021 | tissue | OX407020 | OX407200 | -- | 9 | 3.5 | 5.5 | 3.2 | 788.90 |
| AMV6 | Amvrakikos Gulf | 38.9856717451461/20.94545047741468 | 17/4/2021 | tissue | OX407021 | OX407201 | -- | 13.3 | 6 | 7.3 | 6 | 1351.85 |
| AMV7 | Amvrakikos Gulf | 38.9856717451461/20.94545047741468 | 17/4/2021 | tissue | OX407022 | OX407202 | -- | 15.1 | 6.4 | 8.7 | 6.8 | 1559.20 |
| AMV8 | Amvrakikos Gulf | 38.9856717451461/20.94545047741468 | 17/4/2021 | tissue | OX407023 | OX407203 | -- | 26 | 11.2 | 14.8 | 9.5 | 1509.46 |
| AMV9 | Amvrakikos Gulf | 38.9856717451461/20.94545047741468 | 17/4/2021 | tissue | OX407024 | OX407204 | -- | 28 | 14.8 | 13.2 | 10.5 | 1066.10 |
| AMV10 | Amvrakikos Gulf | 38.9856717451461/20.94545047741468 | 17/4/2021 | tissue | OX407025 | OX407205 | -- | 15.8 | 8 | 7.8 | 5.7 | 526.72 |
| AMV11 | Amvrakikos Gulf | 38.9856717451461/20.94545047741468 | 17/4/2021 | tissue | OX407026 | OX407206 | -- | 17.8 | 7.8 | 10 | 7.6 | 1279.47 |
| AMV12 | Amvrakikos Gulf | 38.9856717451461/20.94545047741468 | 17/4/2021 | tissue | OX407027 | OX407207 | -- | 19 | 5.9 | 13.1 | 7.2 | 356.04 |
| AMV13 | Amvrakikos Gulf | 38.9856717451461/20.94545047741468 | 17/4/2021 | tissue | OX407028 | OX407208 | -- | 19.7 | 8.1 | 11.6 | 9.2 | 353.83 |
| AMV14 | Amvrakikos Gulf | 38.9856717451461/20.94545047741468 | 17/4/2021 | tissue | OX407029 | OX407209 | -- | 21.8 | 11.8 | 10 | 9.2 | 1705.51 |
| AMV15 | Amvrakikos Gulf | 38.9856717451461/20.94545047741468 | 17/4/2021 | tissue | OX407030 | OX407210 | -- | 20.2 | 9.2 | 11 | 8.8 | 870.88 |
| AMV16 | Amvrakikos Gulf | 38.9856717451461/20.94545047741468 | 17/4/2021 | tissue | OX407031 | OX407211 | -- | 22.2 | 7.2 | 15 | 7.6 | 845.12 |
| MYT1 | Kalloni (Lesvos) | 39.081244/26.07566 | 19/1/2019 | tissue | OX407032 | OX407212 | 5-6 | 25.8 | 11.2 | 14.6 | 3.5 | 3778.91 |
| MYT2 | Kalloni (Lesvos) | 39.081244/26.07566 | 19/1/2019 | tissue | OX407033 | OX407213 | 5-6 | 30.5 | 13.1 | 17.4 | 4.2 | 1445.67 |
| MYT3 | Kalloni (Lesvos) | 39.081244/26.07566 | 19/1/2019 | tissue | OX407034 | OX407214 | 5-6 | 34.5 | 13.6 | 20.9 | 4.4 | 4144.35 |
| MYT4 | Kalloni (Lesvos) | 39.081244/26.07566 | 19/1/2019 | tissue | OX407035 | OX407215 | 5-6 | 39 | 14 | 25 | 4.5 | 3692.65 |
| MYT5 | Kalloni (Lesvos) | 39.081244/26.07566 | 19/1/2019 | tissue | OX407036 | OX407216 | 5-6 | 27.8 | 12.9 | 14.9 | 4 | 3302.31 |
| MYT6 | Kalloni (Lesvos) | 39.081244/26.07566 | 19/1/2019 | tissue | OX407037 | OX407217 | 5-6 | 30.1 | 12.6 | 17.5 | 4.2 | 2625.99 |
| MYT7 | Kalloni (Lesvos) | 39.081244/26.07566 | 19/1/2019 | tissue | OX407038 | OX407218 | 5-6 | 34.2 | 12.8 | 21.4 | 4.3 | 3051.15 |
| MYT8 | Kalloni (Lesvos) | 39.081244/26.07566 | 19/1/2019 | tissue | OX407039 | OX407219 | 5-6 | 30.5 | 12.4 | 18.1 | 3.8 | 3265.91 |
| MYT9 | Kalloni (Lesvos) | 39.081244/26.07566 | 19/1/2019 | tissue | OX407040 | OX407220 | 5-6 | 29.3 | 11.4 | 17.9 | 3.5 | 3370.44 |
| TS4 | Kalloni (Lesvos) | 39.20174957104768/26.24919936396623 | 12/2018 | tissue | OX407044 | OX407224 | 1–1.5 | -- | -- | -- | -- | 464.10 |
| TS5 | Kalloni (Lesvos) | 39.20174957104768/26.24919936396623 | 12/2018 | tissue | OX407045 | OX407225 | 1–1.5 | -- | -- | -- | -- | 342.85 |
| TS6 | Kalloni (Lesvos) | 39.20174957104768/26.24919936396623 | 12/2018 | tissue | OX407046 | OX407226 | 1–1.5 | -- | -- | -- | -- | 333.15 |
| TS1 | Gera (Lesvos) | 39.062908364108715/26.519669291535266 | 12/2018 | tissue | OX407041 | OX407221 | 2–5 | -- | -- | -- | -- | 380.63 |
| TS2 | Gera (Lesvos) | 39.062908364108715/26.519669291535266 | 12/2018 | tissue | OX407042 | OX407222 | 2–5 | -- | -- | -- | -- | 372.16 |
| TS3 | Gera (Lesvos) | 39.062908364108715/26.519669291535266 | 12/2018 | tissue | OX407043 | OX407223 | 2–5 | -- | -- | -- | -- | 258.47 |
| D1 | Diafani (Karpathos) | 35.762570/27.211337 | 11/7/2018 | eDNA | OX407047 | OX407227 | 13 | 26.5 | 19.7 | 6.8 | 13.6 | 154.00 |
| D2 | Diafani (Karpathos) | 35.762570/27.211337 | 11/7/2018 | eDNA | OX407048 | OX407228 | 13 | 26.3 | 17.1 | 9.2 | 15.6 | 231.97 |
| D3 | Diafani (Karpathos) | 35.762570/27.211337 | 11/7/2018 | eDNA | OX407049 | OX407229 | 13 | 32.1 | 23.4 | 8.7 | 15.1 | 201.74 |
| D4 | Diafani (Karpathos) | 35.762570/27.211337 | 11/7/2018 | eDNA | -- | -- | 13 | 34.3 | 18.6 | 15.7 | 14.2 | 119.36 |
| GD1 | Diafani (Karpathos) | 35.762570/27.211337 | 14/7/2018 | eDNA | OX407050 | OX407230 | 13 | 29.9 | 19.7 | 10.2 | 14.9 | 259.00 |
| GD2 | Diafani (Karpathos) | 35.762570/27.211337 | 14/7/2018 | eDNA | OX407051 | OX407231 | 13 | 23.2 | 15.4 | 7.8 | 12.3 | 216.09 |
| GD3 | Diafani (Karpathos) | 35.762570/27.211337 | 14/7/2018 | eDNA | OX407052 | OX407232 | 13 | 26.4 | 19.1 | 7.3 | 13.2 | 197.67 |
| GD4 | Diafani (Karpathos) | 35.762570/27.211337 | 14/7/2018 | eDNA | OX407053 | OX407233 | 13 | 23.6 | 15.2 | 8.4 | 11.3 | 202.29 |
| GD5 | Diafani (Karpathos) | 35.762570/27.211337 | 14/7/2018 | eDNA | -- | -- | 13 | 38.5 | 27.1 | 11.4 | 15.7 | -- |
| GD6 | Diafani (Karpathos) | 35.762570/27.211337 | 14/7/2018 | eDNA | OX407054 | OX407234 | 13 | 45.1 | 30.2 | 14.9 | 19.3 | 167.51 |
| GD7 | Diafani (Karpathos) | 35.762570/27.211337 | 14/7/2018 | eDNA | OX407055 | -- | 13 | 26.9 | 18.2 | 8.7 | 14.7 | 180.83 |
| GD8 | Diafani (Karpathos) | 35.762570/27.211337 | 14/7/2018 | eDNA | OX407056 | OX407235 | 13 | 33.6 | 23.4 | 10.2 | 13.5 | 215.06 |
| GD9 | Diafani (Karpathos) | 35.762570/27.211337 | 14/7/2018 | eDNA | -- | -- | 13 | 29.8 | 20 | 9.8 | 14.6 | 290.29 |
| ID1 | Diafani (Karpathos) | 35.762570/27.211337 | 14/7/2018 | eDNA | OX407060 | OX407239 | 13 | 26 | 18 | 8 | 16 | 172.16 |
| ID2 | Diafani (Karpathos) | 35.762570/27.211337 | 14/7/2018 | eDNA | -- | -- | 13 | 26 | 16 | 10 | 13 | 439.95 |
| ID3 | Diafani (Karpathos) | 35.762570/27.211337 | 14/7/2018 | eDNA | -- | -- | 13 | 43 | 26 | 17 | 20 | -- |
| ID4 | Diafani (Karpathos) | 35.762570/27.211337 | 14/7/2018 | eDNA | OX407061 | OX407240 | 13 | 18 | 11 | 7 | 10 | 214.66 |
| ID5 | Diafani (Karpathos) | 35.762570/27.211337 | 14/7/2018 | eDNA | OX407062 | OX407241 | 13 | 18 | 12 | 6 | 10 | 147.50 |
| ID6 | Diafani (Karpathos) | 35.762570/27.211337 | 14/7/2018 | eDNA | -- | -- | 13 | 33 | 18 | 15 | 17 | 126.57 |
| ID7 | Diafani (Karpathos) | 35.762570/27.211337 | 14/7/2018 | eDNA | OX407063 | OX407242 | 13 | 39 | 26 | 13 | 18 | 365.58 |
| Χ1 | Tristomo (Karpathos) | 35.820845/27.211023 | 8/7/2018 | eDNA | OX407007 | OX407188 | 6 | 39.5 | 24.4 | 15.1 | 17.7 | 265.69 |
| Χ2 | Tristomo (Karpathos) | 35.820845/27.211023 | 8/7/2018 | eDNA | OX407008 | OX407189 | 6 | 62.5 | 50.2 | 12.3 | 27.6 | 266.82 |
| Χ3 | Tristomo (Karpathos) | 35.820845/27.211023 | 8/7/2018 | eDNA | OX407009 | OX407190 | 6 | 49.5 | 29 | 20.5 | 21 | 204.00 |
| Χ4 | Tristomo (Karpathos) | 35.820845/27.211023 | 8/7/2018 | eDNA | OX407010 | -- | 6 | 53.1 | 30.2 | 22.9 | 22.7 | 210.37 |
| Χ5 | Tristomo (Karpathos) | 35.820845/27.211023 | 8/7/2018 | eDNA | -- | -- | 6 | 42.4 | 19.2 | 23.2 | 18.1 | 125.13 |
| Χ6 | Tristomo (Karpathos) | 35.820845/27.211023 | 8/7/2018 | eDNA | OX407011 | OX407191 | 6 | 51.6 | 27.2 | 24.4 | 23.9 | 168.71 |
| Χ7 | Tristomo (Karpathos) | 35.820845/27.211023 | 8/7/2018 | eDNA | OX407012 | OX407192 | 6 | 47.3 | 20.9 | 26.4 | 21.2 | 247.95 |
| Χ8 | Tristomo (Karpathos) | 35.820845/27.211023 | 8/7/2018 | eDNA | OX407013 | OX407193 | 6 | 23.9 | 13.1 | 10.8 | 14.6 | 290.13 |
| Χ9 | Tristomo (Karpathos) | 35.820845/27.211023 | 8/7/2018 | eDNA | OX407014 | OX407194 | 6 | 27.6 | 20.4 | 7.2 | 15.4 | 114.81 |
| Χ10 | Tristomo (Karpathos) | 35.820845/27.211023 | 8/7/2018 | eDNA | OX407015 | OX407195 | 6 | 43.2 | 19.4 | 23.8 | 20.2 | 468.92 |
| GT1 | Tristomo (Karpathos) | 35.820845/27.211023 | 13/7/2018 | eDNA | OX407057 | OX407236 | 6 | 53.9 | 24.2 | 29.7 | 20.4 | 57.28 |
| GT2 | Tristomo (Karpathos) | 35.820845/27.211023 | 13/7/2018 | eDNA | -- | -- | 6 | 40.5 | 20.8 | 19.7 | 17.6 | 252.39 |
| GT3 | Tristomo (Karpathos) | 35.820845/27.211023 | 13/7/2018 | eDNA | OX407058 | OX407237 | 6 | 36 | 20.4 | 15.6 | 17.3 | 124.82 |
| GT4 | Tristomo (Karpathos) | 35.820845/27.211023 | 13/7/2018 | eDNA | -- | -- | 6 | 21.5 | 11.2 | 10.3 | 13.1 | 36.91 |
| GT5 | Tristomo (Karpathos) | 35.820845/27.211023 | 13/7/2018 | eDNA | -- | -- | 6 | 29.1 | 16.7 | 12.4 | 16.8 | 68.88 |
| GT6 | Tristomo (Karpathos) | 35.820845/27.211023 | 13/7/2018 | eDNA | -- | -- | 6 | 37.4 | 20.7 | 16.7 | 18.7 | 130.87 |
| GT7 | Tristomo (Karpathos) | 35.820845/27.211023 | 13/7/2018 | eDNA | OX407059 | OX407238 | 6 | 31.8 | 20.6 | 11.2 | 17.8 | 155.51 |
| GT8 | Tristomo (Karpathos) | 35.820845/27.211023 | 13/7/2018 | eDNA | -- | -- | 6 | 8 | 4.4 | 3.6 | 3.3 | 29.88 |
| GT9 | Tristomo (Karpathos) | 35.820845/27.211023 | 13/7/2018 | eDNA | -- | -- | 6 | 39.2 | 21.9 | 17.3 | 16.7 | 1091.81 |
| IT1 | Tristomo (Karpathos) | 35.820845/27.211023 | 13/7/2018 | eDNA | -- | -- | 6 | 18 | 6 | 12 | 11.5 | 222.31 |
| IT2 | Tristomo (Karpathos) | 35.820845/27.211023 | 13/7/2018 | eDNA | OX407064 | OX407243 | 6 | 33 | 21 | 12 | 18 | 254.18 |
| IT3 | Tristomo (Karpathos) | 35.820845/27.211023 | 13/7/2018 | eDNA | -- | -- | 6 | 34 | 18 | 16 | 16.5 | 266.81 |
| IT4 | Tristomo (Karpathos) | 35.820845/27.211023 | 13/7/2018 | eDNA | -- | -- | 6 | 39 | 18.5 | 20.5 | 21.5 | 241.08 |
| IT5 | Tristomo (Karpathos) | 35.820845/27.211023 | 13/7/2018 | eDNA | -- | -- | 6 | 27.5 | 11 | 16.5 | 15.5 | 188.90 |
| IT6 | Tristomo (Karpathos) | 35.820845/27.211023 | 13/7/2018 | eDNA | -- | -- | 6 | 30.5 | 16 | 14.5 | 17.5 | -- |
| IT7 | Tristomo (Karpathos) | 35.820845/27.211023 | 13/7/2018 | eDNA | -- | -- | 6 | 36.5 | 18 | 18.5 | 19.5 | 293.50 |
| IT8 | Tristomo (Karpathos) | 35.820845/27.211023 | 13/7/2018 | eDNA | OX407065 | OX407244 | 6 | 53 | 26 | 27 | 23 | 217.03 |
| IT9 | Tristomo (Karpathos) | 35.820845/27.211023 | 13/7/2018 | eDNA | -- | -- | 6 | 40 | 23 | 17 | 15.5 | 282.46 |
| IT10 | Tristomo (Karpathos) | 35.820845/27.211023 | 13/7/2018 | eDNA | -- | -- | 6 | 42 | 23 | 19 | 21 | 179.44 |
| IT11 | Tristomo (Karpathos) | 35.820845/27.211023 | 13/7/2018 | eDNA | OX407066 | -- | 6 | 49.5 | 27.5 | 22 | 22 | 346.87 |
| IT12 | Tristomo (Karpathos) | 35.820845/27.211023 | 13/7/2018 | eDNA | OX407067 | OX407245 | 6 | 48 | 24.5 | 23.5 | 17 | 195.00 |
| IT13 | Tristomo (Karpathos) | 35.820845/27.211023 | 13/7/2018 | eDNA | OX407068 | OX407246 | 6 | 13 | 13 | 0 | 11 | 159.36 |
| IT14 | Tristomo (Karpathos) | 35.820845/27.211023 | 13/7/2018 | eDNA | -- | -- | 6 | 43 | 23 | 20 | 17.5 | 183.28 |
| LAV01 | Lavrio (Attica) | 37.757668/24.077698 | 3/8/2018 | tissue | OX406989 | OX407247 | 12 | 33 | 17 | 16 | 16.7 | 1951.75 |
| LAV02 | Lavrio (Attica) | 37.757373/24.077915 | 3/8/2018 | tissue | OX406990 | OX407248 | 14 | 53.8 | 34.6 | 19.2 | 21.2 | 1130.01 |
