# Supplementary material for: Insights on Pinna nobilis population genetic structure in the Aegean and Ionian Sea
Source: PeerJ. 2023 Nov 29;11:e16491. doi: 10.7717/peerj.16491 (PMC10693241; doi:10.7717/peerj.16491)
Supplement: Supplemental Information 10 [file peerj-11-16491-s010.docx]

Supplementary Table 3: The linear regression equations for the different study sites and the shell dimensions of the individuals.

|  | **Study sites** | **Linear equation** | **R^2^** |
| --- | --- | --- | --- |
| Height inside the sediment - Height above sediment | All study sites | y = 0.5978x + 9.4007 | 0.1641 |
|  | Crete | y = 2.2945x - 12.61 | 0.4914 |
|  | Attica | y = 2.881x - 18.661 | 0.625 |
|  | Chalkidiki | - | - |
|  | Lesvos | y = 0.1908x + 9.1115 | 0.4708 |
|  | Karpathos | y = 0.5941x + 10.877 | 0.26 |
|  | Amvrakikos | y = -0.513x + 13.799 | 0.2309 |
| Greater width - Total height | All study sites | y = 1.696x + 8.565 | 0.6436 |
|  | Crete | y = 3.5383x - 20.346 | 0.8835 |
|  | Attica | y = 3.5292x - 19.923 | 0.9112 |
|  | Chalkidiki | - | - |
|  | Lesvos | y = 8.628x - 3.5956 | 0.6453 |
|  | Karpathos | y = 2.3778x - 4.6216 | 0.8684 |
|  | Amvrakikos | y = 2.3491x + 0.8066 | 0.8231 |
