# Supplementary material for: Insights on Pinna nobilis population genetic structure in the Aegean and Ionian Sea
Source: PeerJ. 2023 Nov 29;11:e16491. doi: 10.7717/peerj.16491 (PMC10693241; doi:10.7717/peerj.16491)
Supplement: Supplemental Information 11 — p-value (population effect on genetic differentiation): 0.01. [file peerj-11-16491-s011.docx]

Supplementary Table 4: Pairwise F_ST_ values comparing populations in the Ionian, North Aegean and South Aegean. p-value (population effect on genetic differentiation): 0.01.

|  | Ionian Sea | North Aegean Sea |
| --- | --- | --- |
| North Aegean Sea | 0.0012 |  |
| South Aegean Sea | 0.0011 | 0.0032 |
