# Supplementary material for: Insights on Pinna nobilis population genetic structure in the Aegean and Ionian Sea
Source: PeerJ. 2023 Nov 29;11:e16491. doi: 10.7717/peerj.16491 (PMC10693241; doi:10.7717/peerj.16491)
Supplement: Supplemental Information 12 — p-value (population effect on genetic differentiation): 0.01. [file peerj-11-16491-s012.docx]

Supplementary Table 5: Pairwise F_ST_ values comparing populations in the Adriatic Sea, Ionian Sea, North Aegean Sea, South Aegean Sea, Levantine Sea, Western Mediterranean. p-value (population effect on genetic differentiation): 0.01.

|  | Adriatic Sea | Western Mediterranean | Ionian Sea | North Aegean Sea | South Aegean Sea |
| --- | --- | --- | --- | --- | --- |
| Western Mediterranean | 0.0295 |  |  |  |  |
| Ionian Sea | 0.0423 | 0.0054 |  |  |  |
| North Aegean Sea | 0.0755 | 0.0320 | 0.0113 |  |  |
| South Aegean Sea | 0.0746 | 0.0282 | 0.0099 | 0.0062 |  |
| Levantine Sea | 0.0131 | 0.0181 | 0.0276 | 0.0686 | 0.0691 |
