# Supplementary material for: Insights on Pinna nobilis population genetic structure in the Aegean and Ionian Sea
Source: PeerJ. 2023 Nov 29;11:e16491. doi: 10.7717/peerj.16491 (PMC10693241; doi:10.7717/peerj.16491)
Supplement: Supplemental Information 13 — p-value (population effect on genetic differentiation): 0.01. [file peerj-11-16491-s013.docx]

Supplementary Table 6: Pairwise F_ST_ values comparing populations in the Adriatic Sea,

Ionian Sea, North Aegean Sea, South Aegean Sea, Levantine Sea, Western Mediterranean,

Tunisian Plateau/Gulf of Sidra. p-value (population effect on genetic differentiation): 0.01.

|  | Ionian Sea | North Aegean Sea | South Aegean Sea | Levantine Sea | Western Mediterranean | Tunisian Plateau/Gulf of Sidra |
| --- | --- | --- | --- | --- | --- | --- |
| North Aegean Sea | 0.0008 |  |  |  |  |  |
| South Aegean Sea | 0.0053 | 0.0038 |  |  |  |  |
| Levantine Sea | 0.0332 | 0.0555 | 0.0608 |  |  |  |
| Western Mediterranean | 0.0002 | 0.0011 | 0.0037 | 0.0374 |  |  |
| Tunisian Plateau/Gulf of Sidra | 0.0031 | 0.0005 | 0.0037 | 0.0698 | 0.0037 |  |
| Adriatic Sea | 0.0235 | 0.0350 | 0.0417 | -0.0058 | 0.0227 | 0.0452 |
